# Sympathetic Ophthalmia

**Published:** 1915-03

**Authors:** 


**Sympathetic Ophthalmia.** Deutschmann, Ophthalmoscope, Nov., 1914, has demonstrated a gram-positive diplococcus or possible sarcina in the human choroid and has produced the disease in rabbits. The course from one to the other eye in the same individual is by way of the lymph spaces communicating at the chiasm. From the choroid, infection may pass directly into the intervaginal space, or along the anterior ciliary vessels around the eye-ball within the muscles and back into the optic nerve lymph-spaces by way of the central vessels. Chronic inflammatory foci in the meninges exist but cause no symptoms.

